# Quercetin and Its Fermented Extract as a Potential Inhibitor of Bisphenol A-Exposed HT-29 Colon Cancer Cells’ Viability

**DOI:** 10.3390/ijms24065604

**Published:** 2023-03-15

**Authors:** Nataly García-Gutiérrez, Gabriel Luna-Bárcenas, Guadalupe Herrera-Hernández, Rocio Campos-Vega, Sara Julietta Lozano-Herrera, Ana Alicia Sánchez-Tusié, Pablo García-Solis, Haydé Azeneth Vergara-Castañeda

**Affiliations:** 1 Advanced Biomedical Research Center, School of Medicine, Universidad Autónoma de Querétaro, Santiago de Querétaro 76140, Mexico; nat_gagu5487@hotmail.com (N.G.-G.); sarajuliettalo@gmail.com (S.J.L.-H.); anatusie@gmail.com (A.A.S.-T.); pablo.garcia@uaq.mx (P.G.-S.); 2 Cinvestav-Centro de Investigación y de Estudios Avanzados del Instituto Politécnico Nacional, Unidad Querétaro, Santiago de Querétaro 76230, Mexico; gluna@qro.cinvestav.mx; 3 Instituto Nacional de Investigaciones Forestales, Agrícolas y Pecuarias, Campo Experimental Bajío INIFAP, Km 6.5 Carretera Celaya San Miguel Allende, Guanajuato 38110, Mexico; lupis_herrera@msn.com; 4 Research and Graduate Studies in Food Science, School of Chemistry, Universidad Autónoma de Querétaro, Santiago de Querétaro 76140, Mexico; chio_cve@yahoo.com.mx

**Keywords:** quercetin, fermented extract of quercetin, bisphenol A, colon cancer

## Abstract

Bisphenol A (BPA) promotes colon cancer by altering the physiological functions of hormones. Quercetin (Q) can regulate signaling pathways through hormone receptors, inhibiting cancer cells. The antiproliferative effects of Q and its fermented extract (FEQ, obtained by Q gastrointestinal digestion and in vitro colonic fermentation) were analyzed in HT-29 cells exposed to BPA. Polyphenols were quantified in FEQ by HPLC and their antioxidant capacity by DPPH and ORAC. Q and 3,4-dihydroxyphenylacetic acid (DOPAC) were quantified in FEQ. Q and FEQ exhibited antioxidant capacity. Cell viability with Q+BPA and FEQ+BPA was 60% and 50%, respectively; less than 20% of dead cells were associated with the necrosis process (LDH). Treatments with Q and Q+BPA induced cell cycle arrest in the G0/G1 phase, and FEQ and FEQ+BPA in the S phase. Compared with other treatments, Q positively modulated *ESR2* and *GPR30* genes. Using a gene microarray of the *p53* pathway, Q, Q+BPA, FEQ and FEQ+BPA positively modulated genes involved in apoptosis and cell cycle arrest; bisphenol inhibited the expression of pro-apoptotic and cell cycle repressor genes. In silico analyses demonstrated the binding affinity of Q > BPA > DOPAC molecules for ERα and ERβ. Further studies are needed to understand the role of disruptors in colon cancer.

## 1. Introduction

Estrogen disruptors are exogenous compounds of natural or synthetic origin that can mimic or interfere with the biosynthesis, transport, metabolism, or excretion of endogenous hormones due to their high affinity for estrogen receptors [1]. For several years, the effects of estrogen disruptors have been studied in various biological models in a physiological and pathological context to evaluate their toxic or beneficial effects on health. Chemically synthesized disruptors include bisphenol A (BPA), which is derived from the condensation of two phenol molecules with an acetone molecule in the presence of hydrochloric acid, and is used in the manufacture of polycarbonate plastics and epoxy resins. BPA has been used in the manufacture of packaging for food and water [2,3]. In this sense, BPA can dissolve from the materials under temperature and pH conditions and can enter the body [4], reach the colon and cause changes there [5]. However, despite the importance that BPA may have in the colon, the toxic effect on this organ has not yet been studied, thus the mechanism of action is not fully understood. Moreover, BPA has been reported to promote cancer development and affect proliferation and migration in sexual and non-sexual organs, mediating this response through the estrogen-dependent ERα and ERβ signaling pathways [6]. BPA acting at nanomolar concentrations on SW480 colon cancer cells modulated 56 proteins involved in cell proliferation and oxidative stress, with increased cell migration and invasion, transition from epithelial to mesenchymal, and increased expression of the transcription factor Snail, a marker of tumor progression [7]. These findings are alarming because BPA is now widely used and its use in food is not properly regulated; this is the case in most countries, including Mexico. Therefore, it is important to search for alternatives that can reduce the toxic effects of BPA on the body. This is the case of polyphenols such as quercetin and its metabolites in the fermentative processes of the colon. Quercetin, also called phytoestrogen, is a naturally occurring disruptor produced by secondary metabolism in plants [8]. It is found in apples, onions, and blueberries, among others [9]. After ingestion quercetin enters the colon, where it is converted into various molecules, including the major component, 3,4-dihydroxyphenylacetic acid (DOPAC) [10]. The observed biological properties of quercetin include anti-inflammatory, antiproliferative, apoptotic and antioxidant effects in in vivo and in vitro models [11,12,13]. Its effect is so strong that it can inhibit the migration and invasion ability in several cancers [14], not only that of the pure molecule but also that of the components that form in the colon after quercetin ingestion [15]. In addition, phytoestrogens can interact with ERα and ERβ by mimicking the action of estradiol and inducing apoptotic cell death. Quercetin can activate p38 in DLD-1 colon cancer cells, which in turn promotes caspase-3 activation, PARP protein cleavage, and consequently, cell death [16]. These studies suggest a possible chemopreventive or therapeutic effect of quercetin. However, further studies are needed to substantiate its effect as a natural disruptor and to establish its predominant effect over BPA toxicity. Therefore, the aim of this study is to elucidate an antiproliferative mechanism of action of quercetin and its fermented colonic extract acting on colon cancer cells co-exposed to BPA.

## 2. Results and Discussions

**Figure 1:**
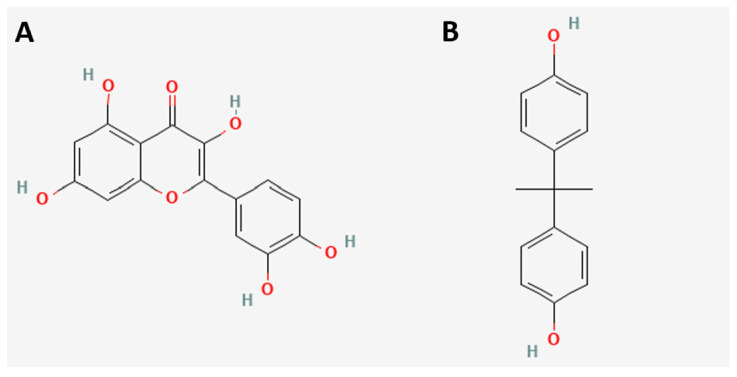
Chemical structures of Q (**A**) and BPA (**B**). The images were downloaded from PubChem, Q = CID: 5,280,343 and BPA = CID: 6623.

The chemical structures of quercetin (Q) and bisphenol A (BPA) are shown in Figure 1. Both molecules contain in their structure aromatic rings and hydroxyls. However, in addition, quercetin contains a carbonyl group and an ether group, which implies the important biological differences mentioned above [7,10].

Gastrointestinal digestion and in vitro fermentation of Q in the colon lead to the formation of several metabolite derivatives. The compounds quantified in the fermented extract of quercetin (FEQ) by HPLC were Q and 3,4-dihydroxyphenylacetic acid (DOPAC) (Table 1).

DOPAC is derived from the metabolism of Q, since a weak peak was observed in the fecal inoculum and in the chromatogram of the fermentation control (FC). Increased incubation time of the in vitro fermentation also increased Q and DOPAC concentrations after 24 h, suggesting that the type of bacteria present in the feces is critical for their metabolism. Examples of bacteria that can convert Q to DOPAC include *Clostridium perfringens* and *Bacteroides fragilis* [17]. In addition, acidification of the medium during the fermentation itself could also cause a change in the Q molecule, making it more soluble and quantifiable by HPLC [18]. In one study, 0.2 µM Q was added to an inoculum of rat feces for 24 h, resulting in 4.7 μM DOPAC [10]. The presence of both Q and DOPAC in 24 h FEQ could confer important biological properties, such as antioxidant capacity. Antioxidant molecules play an important role in protecting against the oxidative damage caused by free radicals [19]. The antioxidant capacity of Q metabolites measured by DPPH was higher than that of pure Q (Table 1). On the other hand, the ORAC assay showed the highest antioxidant capacity of FEQ compared with Q. The difference between the results of the two methods might be because the ORAC reagent measures the scavenging activity of the antioxidant toward the peroxyl radical 2,2′-azinobis(2-amidinopropane) dihydrochloride using fluorescein, a fluorescent probe, indicating the ability of the compounds to neutralize free radicals by donating hydrogen atoms, whereas DPPH involves electron transfer. The loss of fluorescence indicates an increase in the oxidizing power of the free radical [20]. In contrast, the DPPH method is based on the reduction of the free radical 2,2-diphenyl-1-picrylhydrazyl in the presence of an antioxidant [21]. This suggests that both pure Q and FEQ, which have significant antioxidant capacity, can trigger important biological processes. Moreover, FC showed low antioxidant capacity, hence the results of FEQ can be attributed to Q and/or metabolites, but not to the components used for its processing. The high capacity of pure Q to neutralize free radicals by donating electrons and protons has already been demonstrated by other authors [22], however, this is the first report on the antioxidant capacity of its fermented extract.

Studies on the effect of Q and BPA were performed on the human colon adenocarcinoma cell line HT-29, which is a powerful tool to study the effect of dietary components on the intestine and complements the in vivo and ex vivo studies [23].

**Figure 2:**
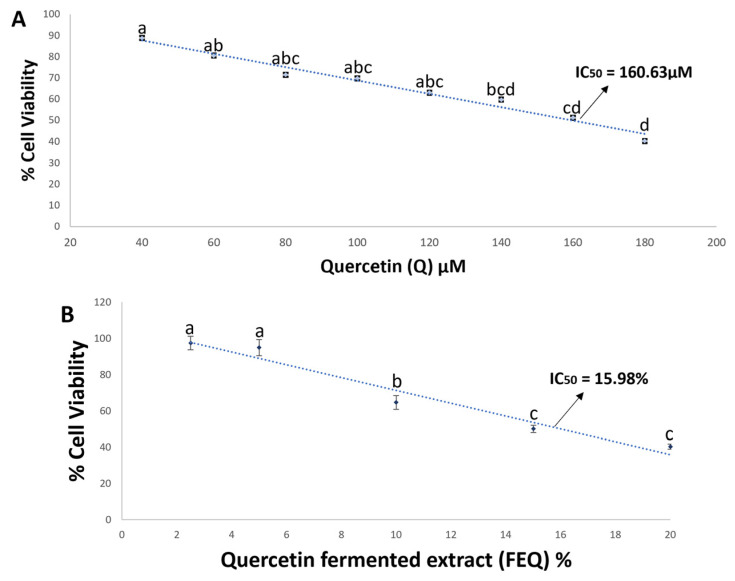
Dose–response curve of Q (**A**) and FEQ (**B**) in HT-29 cells after 48 h of treatment. Each value represents the average of three independent experiments in triplicate ± ES. Different letters indicate significant difference (*p* < 0.05) by Tukey’s test. Q-treated cells were normalized to the negative control. The median inhibitory concentration or IC_50_ refers to the necessary concentration of the treatment to inhibit the survival of 50% of the cell population subjected to the treatment.

In the present study, administration of pure Q or its fermented extract to colon carcinoma cell line HT-29 for 48 h dose-dependently decreased cell viability (Figure 2A,B).

The IC_50_ of Q and FEQ were 160.63 µM and 15.98%, respectively. The amount of Q in the IC_50_ of FEQ is equivalent to 7.48 µM. However, different metabolites are present in FEQ, suggesting a synergistic activity in the antiproliferative effect of the fermentation extract. DOPAC (1 µM) reduced the viability of HT-29 cells by 50% [15], and a higher concentration of DOPAC (2.6 µM) reduced the viability of RKO colon cancer cells after 72 h of incubation [24]. The same antiproliferative effect was observed by Gao et al., after 24 h of incubation with 100µΜ DOPAC in HCT116 cells [25], and after 72 h of incubation with a concentration of 50µΜ DOPAC in HT-29 cells [26]. The antioxidant capacity results suggest a possible contribution to the anticancer effect of both treatments, as both Q and FEQ act by donating electrons and protons and may enhance the activity of endogenous antioxidant enzymes. In this way, the oxidative stress characteristic of cancer cells is reduced, which would lead to programmed cell death.

**Figure 3:**
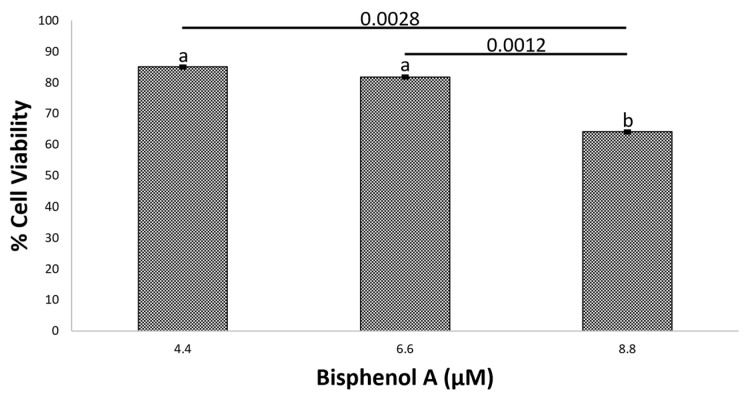
Effect of BPA on HT-29 cells after 48 h treatment. Each value represents the average of three independent experiments in triplicate ± ES. Different letters indicate significant difference (*p* < 0.003) by Tukey’s test. BPA-treated cells were normalized to the negative control.

The viability results of HT-29 cells exposed to BPA are presented in Figure 3.

**Figure 4:**
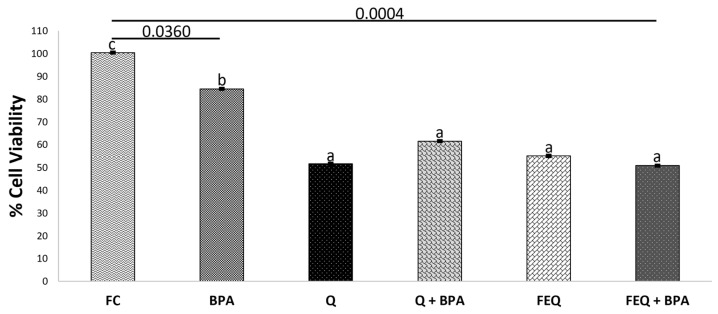
Effect of quercetin and FEQ on HT-29 colon cancer cells treated with BPA. Concentration of treatments: Q 160 μM, BPA 4.4 μM, FEQ 16%. FC: fermentation control. Each value represents the average of three independent experiments in triplicate ± ES, of cells treated with quercetin or FEQ after 48 h. Different letters per column indicate significant difference (*p* < 0.05) by Tukey’s test. The treated cells were normalized with the negative control.

The decrease in cell viability using a concentration of 8.8 µM of BPA could be due to the toxic effect of BPA on the cell, leading to uncontrolled death, known as necrosis. Previous studies have reported the toxicity of BPA in cancer cells, including colon cells, when the concentration of the disruptor increases [27]. In this study, the FDA-approved tolerable dose of BPA (4.4 µM) was used. At this concentration, cell viability decreased by approximately 10%. The process of cell necrosis will be discussed later based on the results of the lactate dehydrogenase assay. Simultaneous exposure of Q to BPA or FEQ to BPA resulted in a percent viability between 50 and 60%, which is very similar to the IC_50_ of Q and FEQ individually (Figure 4).

Moreover, FC does not reduce cell viability. Thus, the results suggest that the antiproliferative effects of Q and FEQ predominate over the BPA-induced molecular effects, as these alone do not significantly reduce viability. To date, there is no evidence of co-exposure of Q or FEQ to BPA in colon tissue. However, the viability of ovarian cancer cells significantly decreased when co-exposed to BPA and the flavonoid genistein at concentrations of 10 µM and 100 µM, respectively [28]. As with FEQ, the inhibitory effect on cell viability can be attributed not only to Q but also to DOPAC. Part of the mechanism that may be involved in the reduction of cell viability by Q or FEQ is again due to antioxidant capacity, but also to cell cycle arrest.

**Figure 5:**
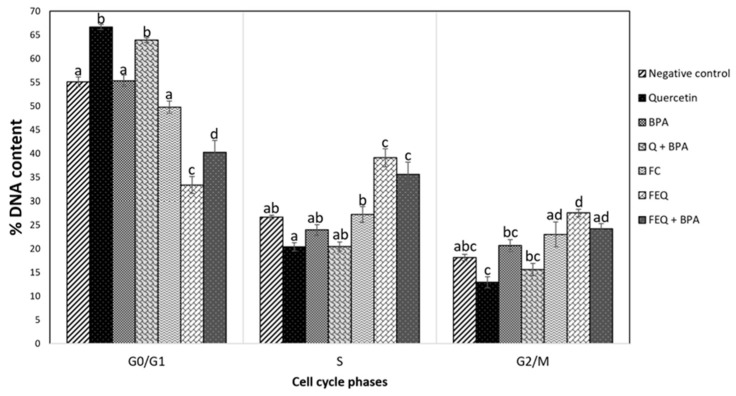
DNA content in the cycle phases of HT-29 cells treated with Q, FEQ and BPA. Concentration of treatments: Q 160 μM, BPA 4.4 μM, FEQ 16%. FC: fermentation control. Each value represents two independent experiments in duplicate ± ES, expressed as percent DNA/500,000 cells. Different letters in a column indicate significant difference between each phase (*p* < 0.05) by Tukey’s test. G_0_: resting phase, G_1_: 1st growth phase, S: DNA synthesis phase, G_2_: 2nd growth phase, M: mitosis phase.

The percentage of cells treated with Q alone or co-exposed to BPA increased in the G0/G1 phase of the cell cycle compared with the other treatments (Figure 5).

In contrast, FEQ alone and co-exposure to BPA reduces the cellular fraction in the G0/G1 phase of the cell cycle compared with the other treatments, but especially compared with FC. Moreover, treatment with FEQ or co-exposure with FEQ+BPA shows a cellular increase in the S phase. The results suggest that Q and FEQ, both alone and with co-exposure to BPA, promote phase-dependent cell cycle arrest followed by cell death. Biologically, this could mean that cancer cells, no longer able to repair the damage caused by Q, initiate their death by apoptosis. Our results are consistent with those of Yang et al., who found that Q at doses of 100 and 200 μM increased the number of cells in the G0/G1 phase of the cell cycle and reduced the S phase of the cycle during 48 h incubation in HT-29 cells [29]. The negative control had no effect on the cell cycle. The effect of BPA was not different from that of the negative control, suggesting that cell death is triggered by other death mechanisms.

**Figure 6:**
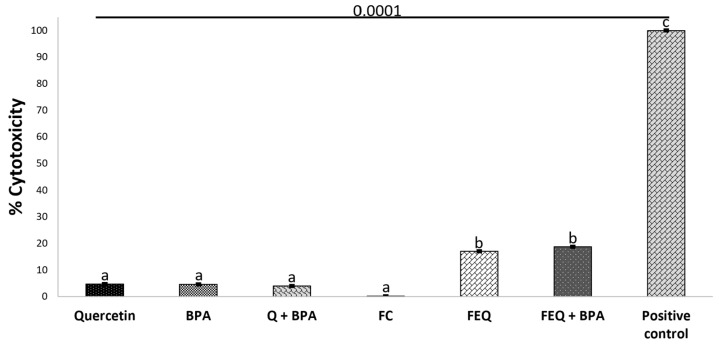
Cytotoxicity (%) effect of Q and FEQ on HT-29 colon cancer cells treated with BPA. Concentration of treatments: Q 160 μM, BPA 4.4 μM, FEQ 16%. FC: fermentation control. Each value represents the average of three independent experiments in triplicate ± ES, from cells treated with quercetin or FEQ after 48 h. Different letters per column express significant difference (*p* < 0.0001) by Tukey’s test. The treated cells were normalized with the negative control. Triton X-100 was used as a positive control.

Necrosis is the death in which the cell lyses in response to intense or prolonged damage and releases its contents into the extracellular space, which in turn triggers inflammatory processes [30]. The necrotic effect of Q alone or in co-exposure to BPA was observed in less than 10% (Figure 6).

Although the percentage of death by necrosis is small, it could contribute to the overall death along with other cell death processes that have not yet been evaluated and could explain the 40% reduction in cell viability by Q, which is different from the result with BPA, because the percentage of necrosis induction is exactly equal to the reduction in cell viability. The percentage of cytotoxicity increased in the positive control due to the lethal damage caused by 1% Triton in the HT-29 cells. Q at a concentration of 100 µM resulted in approximately 5% death by necrosis in 24 h exposure of HT-29 cells [31]. Moreover, the percentage of necrotic cells was dependent on the concentration and exposure time. FEQ and its co-exposure to BPA showed a cytotoxicity of 15%, which does not explain the 35% decrease in cell viability analyzed above, and showed that death was clearly due to Q and its metabolites. The fermentation blank did not trigger this response.

**Figure 7:**
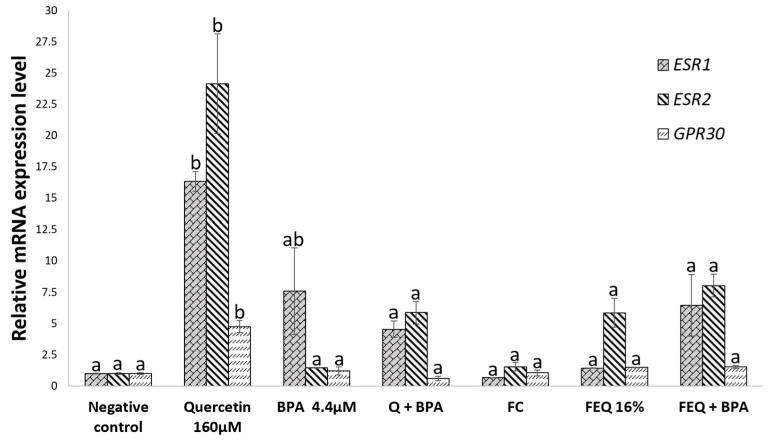
Effect of Q and FEQ co-exposed with BPA on the expression of estrogen receptors α, β and *GPR30* on HT-29 colon cells. Concentration of treatments: Q 160 μM, BPA 4.4 μM, FEQ 16%. FC: fermentation control. Each value represents the mean of a duplicate experiment ± ES. Different letters indicate significant differences between treatments of each gene (*p* < 0.04) by Tukey’s test. The expression of the treatments was normalized with the housekeeping gene actin.

We evaluated the expression of genes involved in different molecular signaling pathways to elucidate the possible mechanisms related to the antiproliferative effect of Q and FEQ treatments. The results of gene expression of *ESR2* and *GPR30* showed no significant difference when compared among groups (Figure 7).

Treatment with Q increased the expression of *GPR30* ˂ *ESR1* ˂ *ESR2* genes (+5, +16 and +24-fold, respectively). Notably, Q significantly increased the expression of *ESR2* and *GPR30* genes compared with the other groups, including FEQ (+6 and +2-fold, respectively). This discrepancy might be due to the exclusive effect of Q on these genes and not to another metabolite present in FEQ, because the concentration of this phytochemical in the fermented extract is low and consequently the genes did not respond to it. Moreover, Q and Q+BPA (*ESR1* by +5-fold and *ESR2* by +6-fold), and FEQ+BPA (*ESR1* by +6-fold and *ESR2* by +8-fold) show a similar trend, suggesting that the treated cells retain the antiproliferative function of *ESR2* over the proliferative function of *ESR1*, even with simultaneous exposure to BPA. The estrogen receptor alpha gene (*ESR1*, *ERα*) is overexpressed in several cancers, including colon cancer, whereas the beta receptor gene is downregulated (*ESR2*, *ERβ*). In HCT8 colon cancer cell line, overexpression of *ESR2* inhibited cell proliferation and increased cell adhesion, suggesting that the possible mechanism of action was to decrease cyclin E, and increase CDK inhibitor P21CIP1, which in turn would block cell cycle progression in the G1-S phase [32]. In addition, the significant decrease in *ESR2* expression was associated with the presence and stage of colorectal polyps and tumors. Barone et al. observed a decrease in *ESR2* gene expression associated with adenomatous tissue with a high degree of dysplasia compared with healthy tissue [33]. However, *ESR2* reduction was accompanied by a decrease in apoptotic cell death and an increase in cell proliferation. Therefore, therapeutic molecules that can promote the expression of this receptor and prevent or promote the progression of colon cancer have been sought. In this sense, treatment with Q may inhibit cell proliferation by increasing the expression of *ESR2* and competing with BPA (+1-fold), although the trend is not statistically significant. Pampaloni et al., showed that polyphenols such as Q and genistein at 50 µM inhibited HCT8 cell proliferation by overexpressing *ESR2* [34]. In another study using *ESR2*-transfected DLD-1 colon cancer cells, 1 µM Q inhibited pro-apoptotic cell growth via activation of P38, caspase-3, and poly(ADP-ribose) cleavage polymerase (PARP) [16]. *ESR2* plays an important role in colon tissue and is therefore considered to be a protective molecule against colon cancer development, and the decrease in *ESR2* expression is inversely proportional to the expression of *ESR1*. Cell proliferation is therefore thought to be the result of a balance between alpha and beta estrogenic receptors [35]. The increased expression of *ESR2* gene compared to *ESR1* in this study may be related to the previously observed decrease in cell viability and cell cycle arrest, which may lead to controlled cell death. However, it must be remembered that the gene transcription rate does not guarantee its translation into a protein, nor that the protein will be bioactive. This is due to several checkpoints that lead to heterogeneity in translation and have shown low correlation between mRNA abundance and protein [36,37]. On the other hand, unlike Q, treatment with BPA increases the *ESR1* expression (+8-fold), which promotes proliferation that is attenuated by Q (+5-fold) or FEQ upon co-exposure. BPA is a molecule that mimics the proliferation of 17β-estradiol in cancer cells by binding to *ESR1*. In MCF-7 cells, BPA at 10 µM increased cell proliferation, increased cyclin D1, E1 and N-cadherin activity, decreased E-cadherin activity, and increased migration capacity, compared to cells to which an estrogen receptor inhibitor was added [38]. On the other hand, BPA at 10 µM can inhibit the activity of the pro-apoptotic proteins caspase-3 and PARP in DLD-1 cells, even in the presence of 17β-estradiol, thus preventing cell death and promoting proliferation [4]. In the present study, it is suggested that Q is a BPA antagonist. There are other phenolic compounds that have been postulated for their potential effects, such as genistein. This molecule has not only exhibited its antiproliferative effect in BG-1 ovarian cancer cells, but it also inhibits cyclin D1 and promotes P21 protein activity, when co-exposed with BPA [28]. For its part, *GPR30* was not modulated in any of the groups except Q, suggesting that *GPR30* is not a target of Q+BPA (+0.6-fold) or FEQ+BPA (+1.5-fold), and may not contribute to the antiproliferative effect.

The study of Q is very extensive. Several mechanisms of action have been proposed to explain the inhibition of cell proliferation, cell cycle arrest and induction of apoptosis. Using the gene array associated with the *p53* pathway, Q induced the expression of the pro-apoptotic genes *APAF1* (+2.28-fold), *FASLG* (+3.23-fold), *KRAS* (+12.43-fold), *TP53AIP1* (+3.23-fold), *TNF* (+3.23-fold) and *TPG3* (+3.23-fold). BPA inhibited the expression of the pro-apoptotic genes *APAF-1* (−29.02-fold), *CASP2* (−2.80-fold), *CASP9* (−3.36-fold), *FADD* (−119.69-fold), *FAS* (−5.53-fold) and *FOXO3* (−5.53-fold) (Table 2).

Like Q, treatment with Q+BPA positively modulated the expression of *FASLG* (+2.45-fold), *KRAS* (+88.50-fold), *TP53AIP1* (+2.47-fold), *TNF* (+2.47-fold) and *TPG3* (+2.47-fold). FEQ induced the expression of *KRAS* (+70.48-fold) and *TNF* (+7.80-fold), whereas FEQ+BPA positively modulated *FASLG* (+5.00-fold), *KRAS* (+75.71-fold), *TP53AIP1* (+5.00-fold), *TNF* (+5.00-fold) and *TPG3* (+5.00-fold). These results suggest that there is some similarity between treatments with Q, FEQ and concomitant exposure to BPA. Although caspase genes were not significantly overexpressed in treatments with Q or FEQ+BPA, this does not mean that they are not biologically relevant. In this sense, *TNF* together with *FASLG* genes may trigger the extrinsic pathway of caspases or the intrinsic pathway by modulating *APAF-1*. On the one hand, the membrane death receptor *TNFR* stimulates the expression of target genes such as *FASL*, which in turn triggers apoptosis after binding to the receptor *FAS* by stimulating the adaptation of *FADD* and the interaction of its N-region terminal with the precursor of caspase-8. Once procaspase-8 is activated by proteolytic degradation, an activation signal for caspase-3 is triggered directly or via the mitochondrial pathway [39]. On the other hand, when the genetic material is damaged, activation of the pro-apoptotic proteins BAX and BAK leads to permeabilization of the outer mitochondrial membrane, which promotes the efflux of proteins, including cytochrome c, into the cytoplasm. This protein, together with apoptotic protease activating factor 1 (APAF-1), forms the apoptosome, which subsequently activates the precursor of caspase-9, which in turn activates caspase-3 [40,41]. Previous studies have shown that Q has an anticancer effect by activating the intrinsic pathway of caspases through the induction of BAX, BAD, cytochrome c, and APAF-1, and inhibiting the expression of cyclin-D1, leading to cell cycle arrest [42]. Furthermore, in a study using LNCaP prostate cancer cells, Q at 100µM was found to decrease phosphorylation of Akt survival proteins and to increase BAX translocation to the mitochondrial membrane, release cytochrome c, activate procaspases-3, -8, and -9, and finally to induce apoptosis. In HeLa cervical cancer cells, Q at 90 µM inhibited the anti-apoptotic expression of Akt and BCL-2, and simultaneously increased mitochondrial cytochrome c levels with depolarization of mitochondrial membrane potential, and caspase-3 activity.

This resulted in decreased cell proliferation, cell cycle arrest in the G2/M phase, and apoptosis [43]. Q at a concentration of 160 µM was able to kill AGS gastric cancer cells by apoptosis, inhibiting MCL-1, BCL-2 and BCL–X, but increasing BAD, BAX and BID [44]. Q has not been studied in detail for the extrinsic pathway of caspases, however, phytochemicals can generally act on FAS, FADD and caspase-8 to induce apoptosis [45]. Q, Q+BPA and FEQ+BPA treatments can induce cell death and the previously observed decline in viability by positively modulating *P53*-independent *TP63* gene expression. Whereas Q and its fermented extract show a pro-apoptotic effect, BPA apparently inhibits it by suppressing *FADD*, *FAS* and intrinsic pathway genes, as well as caspases *CASP2* and *CASP9* and *APAF*-1. These results suggest that the global effect caused by the induction of pro-apoptotic genes and/or inhibition of anti-apoptotic genes by Q and FEQ may produce the opposite effect of BPA when exposed simultaneously. Since BPA has been poorly studied in the colon as much as in reproductive organs, it had anti-apoptotic effects by reducing the expression of *P53*, *P21*, and *BAX* genes in the T47D breast cancer cell line [46]. On the other hand, Q negatively modulated the promoter of cell cycle progression gene *CDKN1A* (−2.02-fold) and positively modulated *EGR1* (+2.62-fold), *GML* (+3.23-fold), *MYOD1* (+3.23-fold), and *TP73* (independently of *P53*) (+3.23-fold). Treatment with Q+BPA induced the expression of *GML* (+2.47-fold), *MYOD1* (+2.47-fold), and *TP73* (+2.47-fold) genes. For its part, FEQ also inhibited cell cycle progression by positively modulating *EGR1* (+2.63-fold), but by co-exposure to BPA, FEQ inhibited *CDKN2A* (−2.68-fold), *E2F3* (−2.15-fold) and *NFKB1* (−2.19-fold) genes and induced *GML* (+5.00-fold), *MYOD1* (+5.00-fold), and *TP73* (+5.00-fold). Modulation of the above genes by treatments with Q, FEQ and their co-exposure to BPA would induce the cell cycle arrest observed in previous results. According to several studies, different colorectal cancer cell lines lead to different cell cycle arrest phases and thus different genes modulated by these phytochemicals. Yang et al. reported arrest in the G0/G1 phase of the HT-29 cell line using 100 to 200 µM Q [29], while the cell increased in the G1 phase in the HCT-116 line using Q and epigallocatechin [47]. In addition, 3,3-dimethylquercetin arrested the cell cycle in G2/M phase in the RKO line by inhibiting genes such as *CDK1*, *CDC25c*, and cyclin D1 [48]. Treatment with BPA prevents cell cycle arrest by inhibiting genes *ATM* (−14.22-fold), *ATR* (−2.62-fold), *BRCA2* (−45.11-fold), *BTG2* (−5.55-fold), *PPM1D* (−4.83-fold), and *PTEN* (−2.32-fold), and allows progression by inducing *CCNH* (+2.26-fold) and *E2F1* (+2.16-fold). As mentioned previously, BPA bypasses apoptosis and promotes cell proliferation through the induction of cyclins, cyclin-dependent kinases, retinoblastoma phosphorylation, and PTEN inhibition, among others [46,49]. Finally, Q can inhibit antigenic processes by inducing the *ADGRB1* gene (+2.18-fold) and preventing the cancer cell from taking up nutrients to survive. It also prevents tumor promotion by negatively modulating *STAT1* (−2.06-fold), positively modulating the tumor suppressor *WT1* (+3.23-fold) and inhibiting transcription by inhibiting gene expression of *TADA3* (−11.94-fold). Q+BPA treatment is comparable to Q in modulating the *WT1* gene. Like Q, FEQ also promotes modulation of the *ADGRB1* (+2.19-fold) and *STAT1* (−2.26-fold) genes, whereas FEQ+BPA induces *ADGRB1* (+2.93-fold) and *WT1* (+5.00-fold) genes. Further studies involving other colorectal cancer cell lines could improve the impact of the results, making them worthwhile for future research.

**Figure 8:**
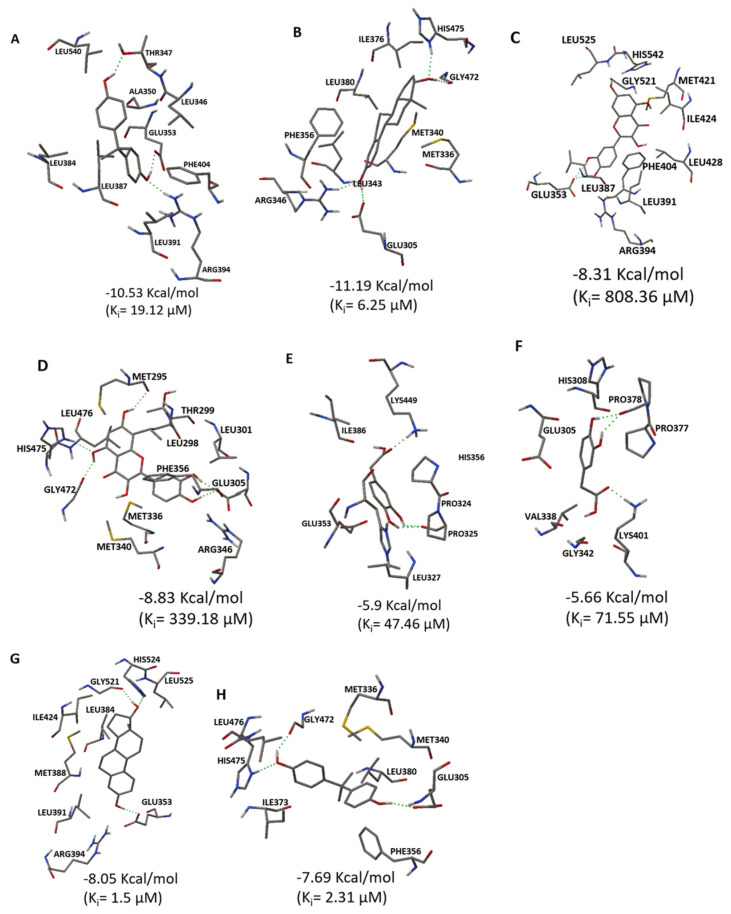
In silico molecular coupling analysis between ligands and receptors. The best coupling bond between (**A**) 17—β estradiol—ERα, (**B**) 17-β estradiol—ERβ, (**C**) Q—ERα, (**D**) Q—ERβ, (**E**) DOPAC—ERα, (**F**) DOPAC—ERβ, (**G**) BPA—ERα, and (**H**) BPA—ERβ. The image represents the interaction between ligands and receptor amino acids. The ΔG values indicate the binding energy expressed in kcal/mol. The inhibition constant (Ki) for each interaction is expressed in μM.

The interaction between the studied ligands (Q and FEQ) and the alpha and beta receptors was analyzed by in silico molecular docking. (Figure 8). The interaction of 17-β estradiol with estrogenic receptors was used as a model for the natural binding of the hormone to these receptors.

In this study, the highest binding affinities for each ligand–protein interaction were selected and the 3D structures of the ligands with the respective amino acids of each receptor were observed. First, the binding affinities range from −11.19 Kcal/mol (Figure 8B) to −5.66 Kcal/mol (Figure 8F). The highest binding affinities correspond to 17-β estradiol and its interaction with both receptors (Figure 8A,B), followed by Q (Figure 8C,D), BPA (Figure 8G,H) and finally DOPAC (Figure 8E,F). 17-β estradiol is the endogenous ligand for estrogen receptors (with −10.53 Kcal/mol for ERα and −11.19 Kcal/mol for ERβ), so the resulting high affinity is not surprising. For this reason, such an interaction can be used for comparison. Q was the ligand with the second highest affinity for both receptors (of −8.21 Kcal/mol for ERα and −8.83 Kcal/mol for ERβ). Its structure interacts with the amino acids leucine 525, 428, 387, 391, histidine 542, glycine 521, methionine 421, isoleucine 424, phenylalanine 404, glutamic acid 353 and arginine 394 of ERα. Particularly the amino acids leucine 525, 391, isoleucine 424, glutamic acid 353, arginine 394, histidine 524 and glycine 521 of ERα that interact with Q also interact with 17-β estradiol, suggesting that both molecules may occupy part of the same active site. As for ERβ, Q interacted with amino acids methionine 295, 336, 340, threonine 299, leucine 301, 298, 476, histidine 475, glycine 472, arginine 346, glutamic acid 305 and phenylalanine 356. In this case, the amino acids interacting with both Q and 17-β estradiol were methionine 336, 340, histidine 475, glycine 472, arginine 345, glutamic acid 305 and phenylalanine 356, with the same active site occupation phenomenon. In addition, the affinity of Q for ERβ is slightly higher than for ERα. This imbalance in favor of ERβ could activate intracellular signals leading to a reduction in cell proliferation. As shown in Figure 7, the induction of ESR2 expression is higher than that of ESR1 because of the Q effect. This, in turn, may activate the expression of genes that promote cell death (Table 2), induce cell cycle arrest in the G0/G1 phase (Figure 5), and consequently reduce viability (Figure 2).

Recently, the interaction of molecules of natural origin with the site of action of estrogen receptors such as 17-β estradiol has been studied. Thus, in an in silico assay, kaempferol interacted with ERα with a binding affinity of −7.0 Kcal/mol, which is lower than that of Q. Moreover, kaempferol interacted with leucine 525, which is consistent with the interaction of Q in this study [50]. In another in silico analysis, the lignans genistein, daidzein, enterodiol, and arctigenin interacted with the ERα amino acids glutamic acid 353, phenylalanine 404, methionine 421, histidine 524, leucine 525, and arginine 394, all of which interact with Q in the present study except for phenylalanine [51]. In contrast, for ERα amino acids and their interaction with DOPAC, only glutamic acid 353 is repeated in its interaction with Q and 17-β estradiol, explaining the low binding affinity of DOPAC for this receptor in contrast to Q and estradiol. This trend is repeated for ERβ, as only glutamic acid 305 interacts with both DOPAC and Q and 17-β estradiol. Although DOPAC does not show high affinity for alpha and beta estrogen receptors, the effect of FEQ on decreased viability (Figure 2B), cell cycle arrest in S and G2/M phase (Figure 5), the tendency for ERβ to increase relative to ERα (Figure 7), and the activation of genes responsible for cell death (Table 2), may be mainly due to Q but also due to the presence of DOPAC. Finally, BPA shows a higher binding affinity for both receptors than DOPAC, but lower than Q and 17-β estradiol. The ERα amino acids that interact with BPA and match Q are phenylalanine 404, leucine 391, arginine 394, leucine 387, and glutamic acid 353. While the amino acids leucine 391, 384, arginine 394, leucine 384, and glutamic acid of ERα match its interaction with 17-β estradiol, meaning that when BPA interacts with ERα, it matches 17-β estradiol in only four amino acids, in contrast to the seven amino acids that match Q and 17-β estradiol. As with ERβ, BPA matches Q in the interaction with glutamic acid 305, methionine 340, 336, phenylalanine 356, leucine 476, glycine 472, and histidine 475; and with 17-β estradiol in amino acids glutamic acid 305, methionine 340, 336, phenylalanine 356, glycine 472, and leucine 380. Therefore, BPA matches 17-β estradiol in six amino acids of ERβ, in contrast to the seven amino acids that match the Q and 17-β estradiol. BPA matches Q in five and seven amino acids in the ERα and ERβ proteins, respectively, suggesting that both molecules compete for the same active site, but that Q shows greater affinity of the compound occupying the site and eliciting the biological response observed in the Q+BPA and FEQ+BPA co-exposure treatments on viability (Figure 2), cell cycle analysis (Figure 5) and cell death gene expression (Table 2). Although in silico analyses are a tool that help to determine the affinity of the ligand–receptor interaction, they have limitations. Therefore, there is a need to develop in vitro experiments that detect molecular activation or inhibition.

**Figure 9:**
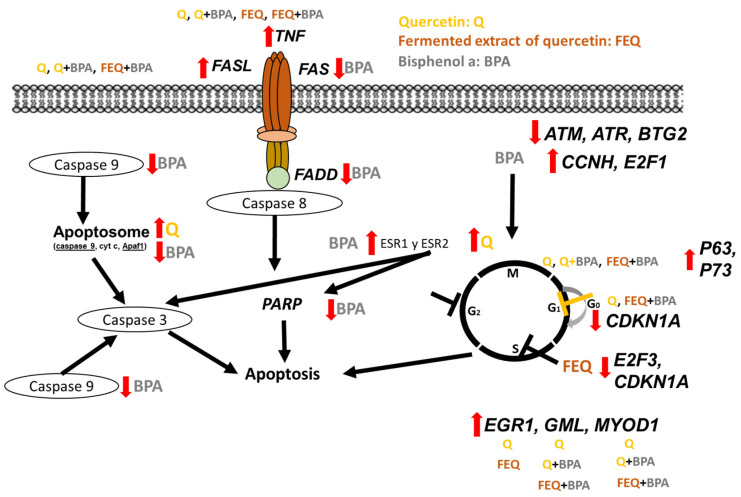
Proposed mechanism of action that explains how Q, FEQ, Q+BPA and FEQ+BPA promote cell inhibition, avoiding the effect of BPA on HT-29 cells. Yellow letters indicate processes carried out by Q, in brown FEQ, and in gray BPA. Red arrows pointing upwards indicate positive regulation and down arrows indicate negative regulation. The highlighted black words correspond to the results found in this study.

The findings obtained in this study suggest a general mechanism of action (Figure 9) that involve several biological processes in HT-29 cells. On the one hand, gene expression results suggest that the treatments might be involved in the intrinsic and extrinsic pathways of caspases; Q, Q+BPA, FEQ and FEQ+BPA promote their activation with subsequent apoptosis, whereas BPA might inhibit both pathways and prevent programmed cell death. Flow cytometry and cell cycle gene expression also supports these findings. Q, Q+BPA, FEQ, and FEQ+BPA prevent cell cycle progression from different points, which could lead to cell inhibition and consequent death; however, BPA appears to have the opposite effect by causing the cell to continue its cycle. This helps us understand how Q and its FEQ have an antiproliferative effect even in the presence of BPA.

## 3. Materials and Methods

The reagents used were obtained from the following suppliers. Quercetin (Q4951) from Sigma-Aldrich^®^, St. Louis, MO, USA, 3,4-dihydroxyphenylacetic acid, 3-(4-hydroxyphenyl) propionic acid, *p*-coumaric acid, 4-methylcatechol, protocatechuic acid, bisphenol A (239658) from Sigma-Aldrich^®^, pepsin, pancreatin, 2,2-diphenyl-1-picrylhydrazyl (DPPH), 6-hydroxy-2,5,7,8-tetramethylchroman-2-carboxylic acid (trolox), 2,2′-azo-bis (2-amidino-propane) dihydrochloride (AAPH), 3-(4,5-dimethylthiazol-2-yl)-2,5-diphenyltetrazolium bromide (MTT, M5655), dimethyl sulfoxide (DMSO, D4540), Triton X-100 (1002214179), from Sigma-Aldrich^®^, hydrogen peroxide, phosphate buffered saline (PBS), ethanol, fetal bovine serum (FBS), and Dulbecco’s modified eagle medium (DMEM, 12800-058) from Gibco^®^.

Commercial kits used were lactate dehydrogenase or LDH (Roche^®^, Basel, Switzerland, 11644793001), Muse Cell Cycle Assay (Merck Millipore^®^, Burlington, MA, USA, MCH100101).

### 3.1. Gastrointestinal Digestion and Colonic Fermentation In Vitro

A previous method was adapted to mimic the physiological gastric conditions from the mouth to the colon in an in vitro system [52]. Quercetin (1 g) was mixed with saliva from healthy volunteers, then the pH was adjusted to 2 and incubated with pepsin for 2 h to simulate the stomach. Later at, the small intestine stage, the pH was adjusted to 7, pancreatin and salts were added, and incubated at 37 °C for 30 min. Thereafter, precipitate (0.5 g) and supernatant (0.5 mL) of the previous solution were mixed with a fecal inoculum obtained from four healthy subjects who had not taken antibiotics for at least three months and had no known gastrointestinal diseases. This final mixture was stored at 37 °C for 6, 12 and 24 h, and considered as fermented extract of quercetin (FEQ). A fermentation control (FC) consisting of distilled water subjected to the same process as quercetin was also included. This study was approved by the Autonomous University of Querétaro. All participants signed the informed consent form.

### 3.2. Analysis of Polyphenols by HPLC-DAD

Polyphenols were analyzed by HPLC (high performance liquid chromatography), coupled with a diode array detector (DAD), in the supernatant of FEQ filtered through a 0.22 µm membrane, according to the method described by Ramírez Jiménez et al. [53]. The FEQ sample was measured at 260, 280, 320 and 340 nm. Commercial standards for quercetin, DOPAC, 3-(4-hydroxyphenyl) propionic acid, *p*-coumaric acid, 4-methylcatechol, and protocatechuic acid were used to establish calibration curves for the detection and quantification of polyphenols. Results were expressed as µg equivalents of standard/µL of sample.

### 3.3. Quantification of Antioxidant Capacity by DPPH and ORAC

Quantification of antioxidant capacity by DPPH assay was proposed by Fukumoto and Mazza, [54]. The mixture of 20 µL FEQ filtrate with 200 µL DPPH was measured at a wavelength of 520 nm in an ELISA spectrophotometer (Thermo scientific^®^, Waltham, MA, USA, multiscan GO). In addition, free radical degradation by the samples was determined using the oxygen radical absorbance capacity (ORAC) test [20]. The FEQ mixture with AAPH in the presence of fluorescein was read in a fluorometer (485 nm excitation/525 nm emission). A calibration curve with Trolox was used for both assays. Results were expressed as equivalent µmol Trolox/µL of sample.

### 3.4. Cell Viability and IC_50_ Determination

The viability of the colon cancer cell line HT-29 obtained from ATCC (HTB-38), was assessed by the colorimetric MTT assay. 5 × 10 ^3^ cells per well were seeded in a sterile 96-well microplate in culture medium supplemented with 10% FBS. After 24 h of incubation, the medium was replaced with different concentrations of quercetin (40–180 μM) or FEQ (5–20%) dissolved in medium supplemented with 5% FBS, and a negative control of cells without treatment was also included. Incubation was again performed for 24 and 48 h. After incubation, the medium was removed and 200 μL of MTT solution was added to each well. Incubation was again performed for 1 h at 37 °C. After this time, the supernatant was removed and 200 μL of DMSO was added to each well and allowed to stand at room temperature for 10 min before reading the values at 540 nm in a spectrophotometer. Percent viability was calculated using the following equation; % Cell viability: (OD_t_/OD_c_) ∗ 100. OD_t_: Average optical density of the treated cultures. OD_c_: Average optical density of the negative control. Percent cell viability was plotted against treatment concentration to determine the average inhibitory dose (IC_50_) by calculating the equation of the straight-line y = mx + b.

### 3.5. Treatments

Cellular variability and determinations were performed under the following treatments dissolved in medium containing 5% FBS: Quercetin at IC_50_, FEQ IC_50_, bisphenol A (4.4 μM, FDA-approved tolerable concentration), quercetin or FEQ mixture with BPA; also included were a negative control, a fermentation control (FC) at 1%, and a Triton X-100 positive control at 1%.

### 3.6. Cytotoxicity Analysis

Cytotoxicity of cells was assessed using the information described in the LDH kit. Cells were seeded in a sterile 96-well microplate (5 × 10 ^3^ cells/well) in culture medium supplemented with 10% FBS. The culture medium was replaced 24 h later with the treatments, including a positive control with 1% Tween X-100. After 48 h of incubation, 50 μL of each treatment was mixed with 100 μL of the LDH reagent and incubated for 15 min at room temperature. The absorbance of the samples was measured using a spectrophotometer at 492 nm. Lactate dehydrogenase activity was calculated using the following equation: %cytotoxicity: (DO_t_ − DOc) * 100/(DO_p_ − DO_c_). DO_t_: Average optical density of treated cultures. OD_c_: Average optical density of negative control. OD_p_: Average optical density of the positive control.

### 3.7. DNA Quantification in the Phases of the Cell Cycle

DNA content in the phases of the cell cycle was quantified using the Muse Cell Cycle kit methodology. Briefly, cells were seeded in a 6-well plate (1 × 10 ^6^ cells/well) in culture medium supplemented with 10% FBS. After 24 h incubation, the medium was replaced with the treatments. After 48 h incubation, the plate was washed with 1 mL of 1X PBS. Cells adhering to the plate were harvested with trypsin. The trypsinized cells were centrifuged at 1800 rpm for 5 min at 30 °C. The supernatant was removed, and the pellet was washed two times with 2 mL of 1X PBS. It was centrifuged again, with the supernatant removed, and the pellet was resuspended in 1 mL of cold 70% ethanol. It was incubated at −20 °C for 3 h. It was then centrifuged at 1800 rpm for 5 min at −4 °C. The pellet was washed twice with 2 mL of PBS 1X, and finally the pellet was resuspended in 20 μL of the reagent and incubated for 30 min. The flow cytometer reading was 5 × 10^5^ cells (Muse™ Cell Analyzer, Millipore, USA). The results were expressed as the percentage of cellular DNA in the three phases of the cell cycle (G0/G1, S, G2/M).

### 3.8. Gene Expression

To determine *ERα*, *ERβ* and *GPR30* expression in the cell lines, total RNA was first extracted from the cells using silica gel membranes from Jena Bioscience GmbH (Jena, Germany). Then, cDNA synthesis was performed using the Script cDNA kit from the Jena Bioscience GmbH (Jena, Germany) under the following conditions: 50 °C for 40 min, and 70 °C for 10 min (Table A1). In addition, amplification conditions were standardized during endpoint PCR using GoTaq Thermo Flexi DNA Polymerase Scientific. The results are considered as potentially modulated sequences if the change between samples of treated and untreated cells was more than 2.0-fold (induction or inhibition). Finally, qPCR assays were performed using the Radiant^TM^ Green Hi-ROX qPCR kit.

### 3.9. Assessment of Gene Expression of the p53 Signaling Pathway

Gene expression was determined using the Human *p53* Signaling Array Pathway (PAHS-027ZA-6 RT^2^ Profiler TM PCR Array, Quiagen) under the conditions described by the supplier. Briefly, total RNA was extracted from treated cells using silica-gel membranes from Jena Bioscience Brand GmbH (Jena, Germany, PP-210S) followed by cDNA synthesis using RT^2^ SYBR Green qPCR Master Mix from Quiagen Sciences, Maryland USA (catalog 330504). The results were analyzed at geneglobe.qiagen.com. Results are considered potentially modulated sequences if the change between samples from treated and untreated cells was more than 2-fold (induction or inhibition).

### 3.10. Molecular In Silico Docking Analysis

To evaluate the interaction between ligands and receptors, molecular in silico docking analysis was performed. The 3D chemical structures of the ligands estradiol (PubChem CID: 5757), quercetin (PubChem CID: 5280343), DOPAC (PubChem CID: 547) and BPA (PubChem: 6623) were obtained from the PubChem database (https://pubchem.ncbi.nlm.nih.gov/, accessed on 22 October 2022). Ligands were created using Discovery Visualizer 19.1.0.18287 (Dassault Syst`emes, V’elizy—Villacoublay, France) and minimized using the RPBS website, AMMOS (Automated Molecular Mechanics Optimization tool for in silico Screening) (https://mobyle.rpbs.univ-paris-diderot.fr/cgi-bin/portal.py, accessed on 22 October 2022). On the other hand, the 3D structures of estrogen receptors alpha (PCG) and beta (5TOA) were downloaded from the Protein DataBank website (https://www.rcsb.org/, accessed on 22 October 2022). In the same way, the receptors were prepared using Discovery Studio Visualizer. The coupling and visualization of the ligands with the receptors was performed using the AutoDockTools version 1.5.7 program after preparing the ligands and receptors as reported by Correa-Basurto et al. [55]. The following equation was used to calculate the values of inhibition constant based on the values of binding energy or Gibbs free energy: ΔG = −RT lnK.

### 3.11. Statistical Analysis

Statistical analysis was performed using JMP software. Results were expressed as mean ± ES. All data were analyzed using ANOVA and the Tukey–Kramer multiple comparison as *pos hoc* (*p* ˂ 0.05).

## 4. Conclusions

This study proves the presence of Q and DOPAC in the colonic fermented extract, which increases the antioxidant capacity. Moreover, DOPAC concentration increased when Q exposure time increased with fecal inoculum, which is due to the metabolization of Q by colonic bacteria. The detection of Q and DOPAC, in addition to other unquantified metabolites, may induce positive biological responses in colon tissues. In this study, the antiproliferative effect of Q and FEQ in colon cancer cells was also observed in the presence of BPA by cellular and molecular studies. Q, FEQ, Q+BPA, and FEQ+BPA decreased cell viability in response to cell cycle arrest, and, to a lesser extent, necrosis. Q plays an important antioxidant and anticancer role, but so do its metabolites produced by bacterial metabolism. To date, however, its involvement in carcinogenesis is unknown, and further studies are needed to investigate its potential health-promoting effects. This study also shows that Q acts at the molecular level, by modulating estrogen genes and the *p53* signaling pathway. The decrease in cell viability may be due to the increase in apoptotic activity caused by the increase in expression of the *ESR2* gene as opposed to the anti-apoptotic *ESR1* gene. This trend is clearly observed in treatments with Q+BPA, FEQ, FEQ+BPA, but not with BPA. Q and FEQ, alone or co-exposed with BPA, generally positively modulate genes exerting pro-apoptotic cell cycle arrest activities, whereas BPA suppresses the expression of pro-apoptotic and cell cycle arrest-promoting genes. Finally, in silico analysis revealed that Q had a higher binding affinity for ERα and ERβ than BPA, whereas DOPAC was the molecule with the lower binding affinity for the receptors. Quercetin showed greater binding affinity for ERβ than for ERα, suggesting that this natural disruptor might induce cell death pathways through its strong interaction with the pro-apoptotic receptor beta and, in turn, prevent BPA from exerting an effect on the anti-apoptotic receptor alpha when both molecules are exposed together. Thus, it can be concluded that Q provides protection against the toxic cellular and molecular effects of BPA. Alternative mechanisms that intervene in the processes triggered by phytochemicals to mitigate the damage caused by disruptors such as BPA remain to be explored.

## Figures and Tables

**Table 1:** Quercetin, polyphenols and antioxidant capacity in the in vitro colonic fermented quercetin extract.

Sample	Quercetin		DOPAC		Antioxidant Capacity	
Fermentation	(µg/µL)	RT	(µg/µL)	RT	DPPH (μmoles/µL)	ORAC (μmoles/µL)
6 h	0.058 a	11.915	0.029 a	6.112	NE	NE
12 h	0.062 a	11.823	0.017 a	6.652	NE	NE
24 h	0.083 b	11.727	0.188 b	6.147	4.316 b	22.130 b
FC	0.052 a	11.470	0.006 a	6.246	0.353 c	2.021 c
Quercetin					5.102 a	21.114 a

FC: fermentation control, DOPAC: 3,4-dihydroxyphenylacetic acid, NE: not evaluated, RT: retention time. Results represent the mean of two duplicate experiments + ES. The antioxidant capacity is expressed as μmol Trolox eq/µL. Each value represents the average of three independent experiments in triplicate ± ES. Different letters per column express significant difference (*p* < 0.05) by Tukey’s test.

**Table 2:** Expression level (fold-change) of genes related to the *p53* signaling pathway of HT-29 cells treated with 160.63 µM Q, 16% FEQ, 4.4 µM BPA, co-treatment Q + BPA or co-treatment FEQ + BPA in relation to negative control cells (up-regulation +, down-regulation −).

Symbol	Description	Q	BPA	Q + BPA	FEQ	FEQ + BPA
		Fold Change				
Genes Associated with Programmed Cell Death (Apoptosis)						
*APAF1*	Apoptotic peptidase activating factor 1 (pro-apoptosis)	**2.28**	**−29.02**	1.52	1.37	−1.57
*BAX*	BCL2-associated X protein (pro-apoptosis)	1.61	1.48	1.07	−1.14	−1.08
*BBC3*	BCL2 binding component 3 (pro-apoptosis)	1.38	−1.15	1.28	−1.42	1.10
*BCL2*	B-cell CLL/lymphoma 2 (anti-apoptosis)	**3.23**	1.33	**2.47**	1.46	**5.00**
*BCL2A1*	BCL2-related protein A1 (anti-apoptosis)	**3.23**	1.33	**2.47**	1.46	**5.00**
*BID*	BH3 interacting domain death agonist (pro-apoptosis)	−1.12	1.40	−1.65	1.07	−1.04
*BIRC5*	Baculoviral IAP repeat containing 5 (anti-apoptosis)	−1.41	**−6.40**	1.19	1.27	1.16
*CASP2*	Caspase-2, apoptosis-related cysteine peptidase (pro-apoptosis)	1.54	**−2.80**	1.16	−1.07	−1.02
*CASP9*	Caspase-9, apoptosis-related cysteine peptidase (pro-apoptosis)	1.67	**−3.36**	1.02	1.16	1.06
*CRADD*	CASP2 and RIPK1 domain containing adaptor with death domain (pro-apoptosis)	−1.04	1.42	−1.36	**−2.08**	1.49
*EI24*	Etoposide induced 2.4 mRNA (pro-apoptosis)	−1.28	−1.75	−1.83	−1.25	−1.47
*FADD*	Fas (TNFRSF6)-associated via death domain (pro-apoptosis)	**−7.83**	**−119.69**	−1.25	−1.21	−1.24
*FAS*	Fas (TNF receptor superfamily, member 6) (pro-apoptosis)	1.34	**−5.53**	1.57	1.22	−1.47
*FASLG*	Fas ligand (TNF superfamily, member 6) (pro-apoptosis)	**3.23**	1.33	**2.47**	1.46	**5.00**
*FOXO3*	Forkhead box O3 (pro-apoptosis)	**−2.59**	**−5.53**	−1.24	−1.04	−1.15
*HK2*	Hexokinase 2 (anti-apoptosis, growth induction)	−1.02	1.00	−1.23	−1.37	−1.32
*IGF1R*	Insulin-like growth factor 1 receptor (anti-apoptosis)	−1.00	1.31	−1.38	−1.57	−1.54
*KRAS*	V-Ki-ras2 Kirsten rat sarcoma viral oncogene homolog (pro-apoptosis)	**12.43**	**40.23**	**88.50**	**70.48**	**75.71**
*MCL1*	Myeloid cell leukemia sequence 1 (BCL2-related) (anti-apoptosis)	1.52	**−2.11**	1.05	1.04	−1.81
*MDM4*	Mdm4 p53 binding protein homolog (mouse) (anti-apoptosis)	−1.14	−1.86	−1.27	−1.16	−1.14
*PIDD1*	P53-induced death domain protein (pro-apoptosis or survival is isoform-dependent)	1.15	−1.29	1.02	−1.35	−1.72
*PRKCA*	Protein kinase C, alpha (regulation of cell proliferation, apoptosis, differentiation, migration, adhesion, angiogenesis)	1.31	−1.38	−1.10	−1.14	−1.31
*SIAH1*	Seven in absentia homolog 1 (Drosophila) (pro-apoptosis)	−1.44	−1.10	−1.50	−1.24	−1.37
*TNF*	Tumor necrosis factor (anti- and pro-apoptosis)	**3.23**	1.33	**2.47**	**7.80**	**5.00**
*TNFRSF10B*	Tumor necrosis factor receptor superfamily, member 10b (pro-apoptosis)	−1.02	1.04	−1.19	1.08	−1.06
*TNFRSF10D*	Tumor necrosis factor receptor superfamily, member 10d, decoy with truncated death domain (anti-apoptosis)	1.62	−1.35	−1.09	−1.25	1.06
*TP53*	Tumor protein p53 (cell cycle regulation and apoptosis)	1.12	1.05	−1.36	−1.75	−1.73
*TP53AIP1*	Tumor protein p53 regulated apoptosis inducing protein 1 (pro-apoptosis)	**3.23**	1.33	**2.47**	1.46	**5.00**
*TP53BP2*	Tumor protein p53 binding protein, 2 (pro-apoptosis)	1.38	1.54	−1.03	−1.12	−1.05
*TP63*	Tumor protein p63 (pro-apoptosis)	**3.23**	1.33	**2.47**	1.46	**5.00**
*TRAF2*	TNF receptor-associated factor 2 (anti-apoptosis)	1.13	1.07	−1.15	−1.20	−1.11
Genes associated with the cell cycle						
*ATM*	Ataxia telangiectasia mutated (cell cycle inhibition)	1.46	**−14.22**	1.37	1.42	−1.18
*ATR*	Ataxia telangiectasia and Rad3 related (cell cycle inhibition)	1.24	**−2.62**	−1.14	1.06	−1.01
*BRCA1*	Breast cancer 1, early onset (cell cycle inhibition)	**−2.67**	1.17	−1.66	−1.35	−1.21
*BRCA2*	Breast cancer 2, early onset (cell cycle inhibition)	**−18.56**	**−45.11**	**−3.04**	−1.24	−1.79
*BTG2*	BTG family, member 2 (cell cycle inhibition)	1.03	**−5.55**	−1.57	−1.44	−1.47
*CCNB1*	Cyclin B1 (cell cycle regulation)	1.54	−1.02	1.05	1.07	1.23
*CCNE1*	Cyclin E1 (cell cycle regulation)	1.19	1.72	−1.20	1.30	1.06
*CCNG1*	Cyclin G1 (cell cycle regulation)	−1.08	1.22	−1.18	−1.66	−1.51
*CCNH*	Cyclin H (cell cycle regulation)	1.64	**2.26**	1.18	1.25	1.21
*CDC25A*	Cell division cycle 25 homolog A (S. pombe) (cell cycle regulation)	−1.24	**−2.06**	−1.80	−1.06	−1.36
*CDC25C*	Cell division cycle 25 homolog C (S. pombe) (cell cycle regulation)	1.37	1.22	1.13	−1.13	1.22
*CDK1*	Cyclin-dependent kinase 1 (cell cycle regulation)	−1.19	**−6.22**	−1.31	−1.32	−1.30
*CDK4*	Cyclin-dependent kinase 4 (cell cycle regulation)	−1.38	1.26	−1.60	−1.70	−1.57
*CDKN1A*	Cyclin-dependent kinase inhibitor 1A (p21, Cip1) (cell cycle regulation)	**−2.02**	**−2.58**	1.24	1.84	**2.08**
*CDKN2A*	Cyclin-dependent kinase inhibitor 2A (melanoma, p16, inhibits CDK4) (cell cycle regulation)	**2.00**	**−9.67**	1.46	1.23	**−2.68**
*CHEK1*	CHK1 checkpoint homolog (S. pombe) (cell cycle regulation)	1.59	**−6.20**	−1.01	1.02	−1.19
*CHEK2*	CHK2 checkpoint homolog (S. pombe) (cell cycle regulation)	1.31	**−2.02**	−1.16	−1.10	−1.22
*E2F1*	E2F transcription factor 1 (cell cycle induction)	1.36	**2.16**	−1.11	1.08	1.01
*E2F3*	E2F transcription factor 3 (cell cycle induction)	−1.41	1.20	−1.74	−1.77	**−2.15**
*EGR1*	Early growth response 1 (regulation of the cell cycle, proliferation, and cell death)	**2.62**	1.99	−1.05	**2.63**	−1.07
*GADD45A*	Growth arrest and DNA-damage-inducible, alpha (cell cycle inhibition)	**−14.67**	1.07	**−3.67**	**−2.49**	−1.90
*GML*	Glycosylphosphatidylinositol anchored molecule-like protein (cell cycle inhibition, pro-apoptosis)	**3.23**	1.33	**2.47**	1.46	**5.00**
*MDM2*	Mdm2 p53 binding protein homolog (mouse) (cell cycle induction)	1.58	−1.65	−1.09	−1.23	−1.09
*MLH1*	MutL homolog 1, colon cancer, nonpolyposis type 2 (*E. coli*) (cell cycle inhibition)	1.60	1.32	−1.02	−1.18	−1.35
*MSH2*	MutS homolog 2, colon cancer, nonpolyposis type 1 (*E. coli*) (cell cycle inhibition)	1.10	1.51	−1.43	−1.03	−1.34
*MYC*	V-myc myelocytomatosis viral oncogene homolog (avian) (cell cycle inhibition)	1.36	**2.06**	1.06	1.06	1.06
*MYOD1*	Myogenic differentiation 1 (cell cycle inhibition)	**3.23**	1.33	**2.47**	1.46	**5.00**
*NF1*	Neurofibromin 1 (cell cycle inhibition, proliferation inhibition, survival inhibition)	1.06	1.30	−1.21	−1.20	−1.32
*NFKB1*	Nuclear factor of kappa light polypeptide gene enhancer in B-cells 1 (cell cycle inhibition, proliferation inhibition, survival inhibition)	−1.03	1.06	−1.57	−1.69	**−2.19**
*PPM1D*	Protein phosphatase, Mg2+/Mn2+ dependent, 1D (cell cycle inhibition)	−1.37	**−4.83**	−1.38	−1.29	−1.42
*PCNA*	Proliferating cell nuclear antigen (cell cycle induction)	1.05	1.31	−1.34	1.06	−1.25
*PRC1*	Protein regulator of cytokinesis 1 (cell cycle induction)	1.46	−1.81	−1.05	−1.12	−1.33
*PTEN*	Phosphatase and tensin homolog (cell cycle inhibition, proliferation inhibition, survival inhibition, migration inhibition)	**−2.12**	**−2.32**	−1.25	−1.27	−1.26
*PTTG1*	Pituitary tumor-transforming 1 (cell cycle induction)	1.89	1.11	1.56	1.52	1.52
*RB1*	Retinoblastoma 1 (cell cycle inhibition)	1.29	1.41	−1.24	−1.66	−1.55
*RPRM*	Reprimo, TP53 dependent G2 arrest mediator candidate (cell cycle inhibition)	1.02	−1.09	−1.79	−1.38	1.13
*TADA3*	Transcriptional adaptor 3 (cell cycle induction, acetylation)	**−11.94**	1.16	1.08	−1.28	−1.19
*TP73*	Tumor protein p73 (pro-apoptosis)	**3.23**	1.33	**2.47**	1.46	**5.00**
Genes associated with angiogenesis, inflammation, autophagy, acetylation, methylation, and tumor suppression						
*ADGRB1*	Brain-specific angiogenesis inhibitor 1 (angiogenesis inhibition)	**2.18**	−1.53	1.32	**2.19**	**2.93**
*DNMT1*	DNA (cytosine-5-)-methyltransferase 1 (methylation)	1.29	1.67	−1.24	−1.01	−1.11
*EGFR*	Epidermal growth factor receptor (proliferation induction)	1.14	1.54	−1.16	−1.29	−1.15
*ESR1*	Estrogen receptor 1 (proliferation induction)	**3.23**	1.77	**2.47**	1.46	**7.16**
*HDAC1*	Histone deacetylase 1 (deacetylation)	1.25	1.42	−1.10	−1.35	−1.13
*IL6*	Interleukin 6 (interferon, beta 2) (pro-inflammation)	**3.23**	1.33	**2.47**	1.46	**5.00**
*JUN*	Jun proto-oncogene (growth regulation)	−1.11	**−4.72**	−1.39	−1.34	−1.93
*KAT2B*	K(lysine) acetyltransferase 2B (acetylation)	−1.21	1.04	−1.09	−1.86	−1.09
*RELA*	V-rel reticuloendotheliosis viral oncogene homolog A (avian) (growth regulation)	1.49	1.68	1.05	1.00	1.09
*SESN2*	Sestrin 2 (autophagy induction)	−1.50	1.09	−1.97	−1.58	−1.67
*SIRT1*	Sirtuin 1 (anti- and pro-tumorogenic)	−1.24	1.67	−1.61	−1.17	−1.04
*STAT1*	Signal transducer and activator of transcription 1, 91kDa (anti- and pro-tumorogenic)	**−2.06**	1.34	−1.92	**−2.26**	−1.72
*TSC1*	Tuberous sclerosis 1 (tumor suppressor)	−1.03	1.19	−1.10	−1.04	−1.11
*WT1*	Wilms tumor 1 (tumor suppressor)	**3.23**	1.33	**2.47**	1.46	**5.00**
*XRCC5*	X-ray repair complementing defective repair in Chinese hamster cells 5 (double-strand-break rejoining) (DNA repair)	−1.72	1.31	−1.03	1.01	−1.02

Bold numbers indicate a ± 2-fold change compared to negative control, using β-Actin, GAPDH, and β-2-microglobulin as housekeeping.

## Data Availability

Not applicable.

## Abbreviations

Q: quercetin, BPA: bisphenol A, DOPAC: 3,4-dihydroxyphenylacetic acid, FEQ: fermented extract of quercetin, FC: fermentation control.

## Appendix A

**Table A1:** Forward and reverse sequences of genes *ERS1*, *ERS2*, *GPR30* and Actin.

Gen	Sequence	Alignment Temperature
ESR1	FWD: TGCTGGCTACATCATCTCGG	60 °C
	REV: CAGGAACTTATCCCTCATATAG	
ESR2	FWD: TCCCACTTCGTAACACTTCCG	64 °C
	REV: ACATTCTATAGCCCTGCTGTGA	
Actin	FWD: ACGGGGTCACCCACACTGTGC	62 °C
	REV: CTAGAAGCATTTGCGGTGGACGATG	
GPR30	FWD: AGTCGGATGTGAGGTTCAG	60 °C
	REV: TCGTGTTGAGGGAGTGCAAG	
